# The appropriateness of single page of activation of the cardiac catheterization laboratory by emergency physician for patients with suspected ST-segment elevation myocardial infarction: a cohort study

**DOI:** 10.1186/1757-7241-19-50

**Published:** 2011-09-12

**Authors:** Soo Hyun Kim, Sang Hoon Oh, Seung Pill Choi, Kyu Nam Park, Young Min Kim, Chun Song Youn

**Affiliations:** 1 Department of Emergency Medicine, College of Medicine, The Catholic University of Korea, Seoul Korea

## Abstract

**Background:**

The early use of reperfusion therapy has a significant effect on the prognosis of patients with ST-segment elevation myocardial infarction (STEMI), and it is recommended that emergency department (ED) physicians activate the cardiac catheterization laboratory (CCL) as soon as possible to treat these patients. The aim of this study was to examine the appropriateness of emergency physician activation of the CCL for patients with suspected STEMI. Inappropriate activations (i.e., false positive activations) were identified according to a variety of criteria.

**Methods:**

All patients with emergency physician CCL activations between August 2009 and April 2011 were included in the study. False positive cases were defined according to ECG criteria and cardiologists' reviews of patients' initial clinical information.

**Results:**

ED physicians used a STEMI page to activate the CCL 117 times. According to reviews by cardiologists, this activation was appropriate 89.8% of the time (in 105/117 cases). Truly unnecessary activation (i.e., cases in which STEMI was not identified by the cardiologists, no clear culprit coronary artery was present, no significant coronary artery disease and cardiac biomarkers were negative) occurred 5.1% of the time (in 6/117 cases).

**Conclusions:**

CCL activation was appropriate for most patients and was unnecessary in a relatively small percentage of cases. This result supports the current recommendation for CCL activation by emergency physicians. Such early activation is a key strategy in the reduction of door-to-balloon time.

## Introduction

Early intervention is fundamental in the treatment of ST-segment elevation myocardial infarction (STEMI), and the timely restoration of coronary blood flow can reduce mortality [1-3]. According to the current American Heart Association (AHA) guidelines for reperfusion, a patient with STEMI should receive fibrinolytics within 30 minutes of arrival (for a 30-minute "door-to-drug" interval) or percutaneous coronary intervention (PCI) within 90 minutes of arrival (for a 90-minute "door-to-balloon" interval) [4]. Several strategies to reduce door-to-balloon time have been recommended, including allowing emergency physicians to bypass routine cardiology consultations and directly activate the cardiac catheterization laboratory (CCL) [5].

If the proportion of false positive CCL activations is acceptably low, this strategy may be the best way to reduce door-to-balloon time. The AHA's STEMI guidelines recommend that emergency physicians make a decision regarding reperfusion therapy within 10 minutes of interpreting a patient's initial electrocardiogram (ECG) [4]. However, in many clinical circumstances, this decision may be challenging due to the lack of previous ECGs, cardiac biomarker results, and serial ST-segment changes. Early activation of the CCL by emergency physicians may be a key strategy in the reduction of door-to-balloon time. Recent evidence suggests that inappropriate, false positive activation is infrequent and occurs between 5.2% and 14% of the time. However, the variation in this range may stem from different definitions of false positive cases [6,7].

The aim of this study was to investigate the appropriateness of emergency physician CCL activation for patients with suspected STEMI. A variety of definitions of false positive cases were used to evaluate this appropriateness.

## Methods

### Settings and patients

This retrospective study was conducted in a tertiary teaching hospital in Seoul, Korea. Seoul St. Mary's Hospital serves a regional population of about 400,000 individuals. The study was approved by the hospital's institutional review board.

In August 2009, new procedures were initiated to reduce door-to-balloon time for STEMI patients at Seoul St. Mary's Hospital. Attending emergency physicians, after reviewing a patient's history and initial ECG, were encouraged to activate the CCL by a single page via the electronic medical record system in cases of suspected STEMI. After this single page, the on-call interventional attending physicians, fellows, and CCL staffs were alerted by text messages on their mobile phones. Text messages included the name, sex, and age of the patient and the admission time (i.e., the door time). The main goals of the STEMI alert system were to reduce door-to-ECG time to 10 minutes and door-to-balloon time to 90 minutes.

All patients who experienced emergency physician activation of the CCL between August 2009 and April 2011 were included in the study. A total of 9 patients were excluded because they were transferred from another hospital after the diagnosis of STEMI (n = 7) or died prior to emergency PCI (n = 2).

### Outcome measures

False positive cases of CCL activation for patients with suspected STEMI were primarily defined according to ECG criteria and a review of initial clinical information. ST elevation was defined as J-point elevation in two or more contiguous leads with a cutoff of greater than or equal to 0.2 mV in V1-V3 and greater than or equal to 0.1 mV in other leads. A left bundle branch block that was not known to be pre-existing was also considered to be a sign of STEMI. The ECGs and initial clinical information for all patients were independently reviewed by 2 cardiologists who were blinded to the patient outcomes. If there were any discrepancies, a third investigator arbitrated these issues. The cardiologists were asked, "if you were in this situation, would you have performed emergency angiography for STEMI?" If the answer was "yes," STEMI was identified.

Other definitions of false positive CCL activation included the absence of a culprit coronary artery, absence of significant coronary artery disease and negative cardiac biomarkers. A culprit coronary artery was defined as the presence of an acute total or subtotal occlusion of a coronary artery or a coronary lesion with a visible thrombus that was responsible for the STEMI. No significant coronary artery disease was defined as less than 50% stenosis in any coronary artery. Positive cardiac biomarkers were defined as elevated troponin I level or a creatine kinase MB fraction peak of greater than 7%.

Truly unnecessary CCL activation was identified when the cardiologists' review did not identify STEMI, the patient did not have a clear culprit coronary artery, significant coronary artery disease was not present and cardiac biomarkers were negative.

A patient's arrival period was categorized as occurring during an on-duty time (Monday to Friday, 8 AM to 6 PM, excluding institutional holidays) or an off-duty time. During off-duty times, the CCL staff would not be routinely available.

### Statistical methods

The distributions of baseline demographics are provided as percentages and means ± standard deviations. In the analysis of patient characteristics and comparison of the STEMI and no STEMI groups, a t-test was used for continuous variables and Fisher's exact test and a chi-squared test were used for categorical variables. Non-normally distributed continuous variables were compared according to median values and tested for statistical significance using the Mann-Whitney test. All statistical analyses were performed using SPSS version 16.0 (SPSS, Chicago, IL), and p values less than or equal to 0.05 were considered significant.

## Results

Not counting excluded patients, between August 2009 and April 2011, emergency department (ED) activation of the CCL by the STEMI page occurred 117 times. During the study period, there were no cases of STEMI in which the emergency physician did not alert the CCL. The baseline demographic characteristics of the patients are shown in Table 1.

**Table 1:** Patient demographics according to ST elevation

	ST elevation, Yes N = 105	ST elevation, No N = 12	p
Sex, male	75 (71.4%)	7 (58.4%)	0.348
Age	63.3 ± 15.4	64.7 ± 16.1	0.777
Chief Complaint			0.161
Chest pain	80 (76.2%)	7 (58.4%)	
Dyspnea	14 (13.3%)	3 (25%)	
Epigastric pain	4 (3.8%)	0 (0%)	
General weakness	3 (2.8%)	0 (0%)	
Syncope	2 (1.9%)	0 (0%)	
Dizziness	1 (1.0%)	0 (0%)	
Palpitation	0 (0%)	1 (8.3%)	
Nausea/Vomiting	1 (1.0%)	1 (8.3%)	
Duty, on	46 (43.8%)	4 (33.3%)	0.487

**Figure 1:**
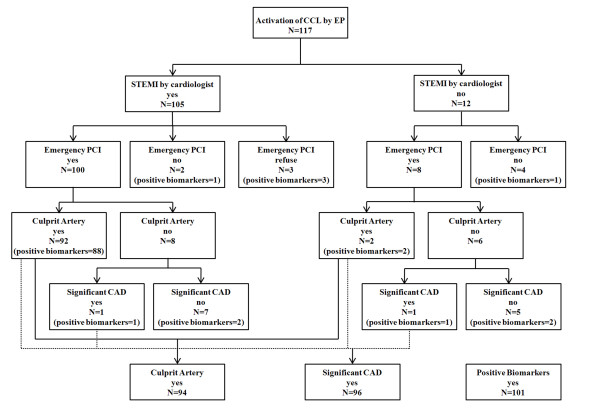
**Flowchart for single page activation of the cardiac catheterization laboratory by emergency physician for patients with suspected ST-segment elevation myocardial infarction**. CCL: cardiac catheterization laboratory, EP: emergency physician, STEMI: ST-elevation myocardial infarction, PCI: percutaneous coronary intervention, CAD: coronary artery disease.

The cardiologists' review determined that 105 of 117 patients (89.8%) had STEMI and of which 2 patients had left bundle branch block. Of these 105 patients, 3 refused emergency coronary angiography due to old age or significant underlying disease, 2 could not receive emergency coronary angiography due to severe congestive heart failure, and 100 underwent emergency coronary angiography. Of those 100 patients, 92 patients had a culprit coronary artery and 93 had significant coronary disease. Eight patients who had no clear culprit coronary artery had the following disorders: variant angina (n = 2), myocarditis (n = 2), chronic renal failure (n = 1), minimal coronary artery disease (n = 1), congestive heart failure (n = 1), and cancer infiltration (n = 1) (Figure 1).

The cardiologists' review determined that 12 patients did not have STEMI. These patients had the following disorders: variant angina (n = 1), unstable angina (n = 2), non-STEMI (n = 3), heart failure (n = 4), 3-vessel disease and referral for coronary artery bypass surgery (n = 1), and minimal coronary artery disease (n = 1). Eight of these 12 patients underwent emergency coronary angiography. Of these, 2 patients had a clear culprit coronary artery and 3 patients had significant coronary artery disease.

The appropriateness of emergency physician CCL activation for patients with suspected STEMI depending on the definition of a false positive were as follows: 89.8% (105/117) of patients were determined by the cardiologists to have STEMI, 82.5% (94/114) had a clear culprit coronary artery, 84.2% (96/114) had significant coronary artery disease and 86.3% (101/117) had positive cardiac biomarkers. Truly unnecessary CCL activation (i.e., when the cardiologists identified no STEMI, no clear culprit coronary artery was present, there was no significant coronary artery disease and cardiac biomarker was negative) occurred for 5.1% (6/117) of patients.

The STEMI group tended to have faster door-to-ECG and door-to-balloon times (Table 2). When the cardinal symptoms were divided according to the presence or absence of chest pain, patients with chest pain were found to have faster door-to-ECG and door-to-balloon times. During on-duty times, the door-to-ECG time was slower, but the door-to-balloon time was faster (Table 3).

**Table 2:** Time intervals according to ST elevation

	ST elevation, Yes N = 105	ST elevation, No N = 12	p
Door-to-ECG time Median, IQR	7 (3, 13)	9.5 (2,17)	0.942
Door-to-balloon time Median, IQR	68 (57, 84)	221 (180, 262)	0.021
% of door-to-balloon time < 90 min	79 (86.8%)	0 (0%)	0.001

**Table 3:** Time intervals according to the chief complaint and on- or off-duty times

	Chief complaint Chest pain N = 87	Chief complaint Other symptoms N = 30	p
Door-to-ECG time Median, IQR	6 (2, 12)	9 (4,16)	0.077
Door-to-balloon time Median, IQR	66.5 (56, 82)	80 (67, 89)	0.028
% of door-to-balloon time < 90 min	65 (85.5%)	14 (82.4%)	0.741
	On duty N = 50	Off duty N = 67	p

Door-to-ECG time Median, IQR	10 (6, 17)	4.5 (1,9)	0.001
Door-to-balloon time Median, IQR	63 (53, 78)	77 (64, 86)	0.013
% of door-to-balloon time < 90 min	38 (88.4%)	41 (82.0%)	0.392

## Discussion

Prompt recognition of STEMI and treatment with early reperfusion therapy can have a significant effect on patient outcomes. [1-3,8]. Several factors can lead to a delay in treatment; these include extended time between the onset of symptoms and the patient's recognition of them, transport to the hospital, and treatment at the emergency department. Delays during in-hospital evaluation can be caused by the "4Ds": door, data (ECG), decisions, and drugs [9]. Bradley et al. have presented several strategies to reduce door-to-balloon time, and one of them is to exclude routine cardiology consultation and have emergency physicians activate the CCL; this strategy could reduce door-to-balloon time by an average of 8.2 minutes [5]. However, some institutions may be resistant to this procedure, especially during off-duty times, out of concern for unnecessary CCL activation.

To assess the appropriateness of CCL activation by emergency department physicians, a clear definition of inappropriate or false positive activation is necessary. Larson et al. defined a false positive as the absence of a clear culprit coronary artery and found that unnecessary CCL activation occurred in 14% of patients [6]. Kontos et al. found that 5.2% of patients had an ECG without ST elevation, did not undergo emergency angiography, and did not have significant coronary artery disease; these patients were identified as cases of unnecessary CCL activation [7].

The ECG is the most immediately accessible and widely used diagnostic tool that guides emergency treatment strategies. An ECG recorded during acute myocardial infarction is of diagnostic, therapeutic, and prognostic significance. However, false positive activation is not synonymous with misinterpretation of an ECG, and in fact, STEMI cannot be definitively diagnosed from an initial ECG. In other words, even when an ECG shows ST elevation, the patient may not be experiencing acute myocardial infarction [10-13]. The standard criteria used to diagnose STEMI include a combination of clinical symptoms, serial ECGs, and serial biomarkers. Unfortunately, the above information is unknown when a patient arrives at the hospital. Therefore, the gold standard definition of a false positive relies on a cardiologist's retrospective determination using limited clinical information and initial ECG findings. Using the reviews of 2 cardiologists, this study found a 10.2% false positive rate; this finding is similar to those of previous studies.

ST-elevation acute coronary syndrome (STE-ACS) results from transmural ischemia typically caused by a fibrin-rich thrombus occluding the infarct-related artery [14]. STE-ACS is classified as an aborted myocardial infarction and as STEMI depending on the presence of myocardial necrosis biomarkers [15]. The MI may be aborted spontaneously before the development of myocardial cell necrosis. Therefore, it is difficult to determine the appropriateness of emergency physician CCL activation with angiographic findings.

Patient care is a hospital's priority, and overtriage is an essential strategy to prevent the catastrophic consequences of undertriage. This lesson can be learned from the trauma system; most Level I trauma centers and trauma specialists consider some degree of overtriage to be necessary to prevent harm to patients [16]. As systems of care are developed for STEMI patients, it is essential that appropriate referrals to STEMI centers and activations of the CCL occur irrespective of final diagnoses.

Our study has several limitations. First, this study presents data from a single tertiary teaching hospital, and the results may not be generalizable. Second, the retrospective nature of the study leaves it vulnerable to several biases. Third, the sample size is relatively small compared to previous studies. Fourth, a cardiologist's ECG reading may not always be accurate. One study found that cardiologists could distinguish between STEMI and non-STEMI with 90% accuracy [17], and another study found they could diagnose STEMI with 75% sensitivity and 85% specificity [18]. This difference may reflect methodological bias. However, from the perspective of systems of care and because there is limited time in which a decision must be made, there may be no better definition of STEMI than a cardiologist's confirmation.

## Conclusion

Approximately 10% of CCL activations were false positives. Truly unnecessary activation was not very high at 7.7%. This result is enough to support current recommendations for CCL activation by emergency physicians; such procedures may be considered a key strategy in the reduction of door-to-balloon time.

## Abbreviations

STEMI: ST-segment elevation myocardial infarction; ED: emergency department; CCL: cardiac catheterization laboratory; AHA: American Heart Association; PCI: percutaneous coronary intervention; ECG: electrocardiogram; EP: emergency physician.

## Competing interests

The authors declare that they have no competing interests.

## Authors' contributions

SHK performed data analysis and drafted the manuscript. SHO acquired data and critical revisions to the manuscript. SPC, KNP, YMK managed the data and critical revisions to the manuscript. CSY conceived the research and drafted the manuscript. Each authors has read and approved the final manuscript.
